# An 8‐week modified FIFA 11+ training program improves postural balance, proprioception and movement performance in Iranian National Ultimate Frisbee players with functional ankle instability

**DOI:** 10.1002/jeo2.70494

**Published:** 2025-10-30

**Authors:** Yeganeh Sharifi, Farideh Babakhani, Ramin Balouchy, Mohamadreza Hatefi

**Affiliations:** ^1^ Department of Sport Injuries and Corrective Exercises, Faculty of Physical Education and Sport Science Allameh Tabataba′i University Tehran Iran

**Keywords:** ankle sprain, dynamic balance, force plate, joint position sense, kinematics

## Abstract

**Purpose:**

Ankle injuries are among the most prevalent injuries experienced by Frisbee players. These injuries can lead to serious consequences, including recurrent sprains, prolonged absences from physical activity, and financial burdens for the players. This underscores the critical need for effective prevention strategies for ankle injuries in Frisbee.

**Method:**

This study aims to evaluate the effectiveness of an 8‐week FIFA 11+ training program on postural balance, proprioception and movement performance in players of the Iranian national Ultimate Frisbee team who exhibit functional ankle instability. In this study, 26 male and female athletes from the national Ultimate Frisbee team, aged between 20 and 30, were selected and randomly divided into experimental and control groups. During the pre‐test phase, postural balance factors, proprioception, and ankle motor function were assessed using a force plate device and Kinovea software. The experimental group then performed modified FIFA 11+ exercises for 8 weeks, after which they were re‐evaluated to assess changes in the aforementioned factors.

**Results:**

A covariance test was employed to analyse the data. The ANCOVA results revealed that the intervention group demonstrated significant improvements in jumping performance (*p* = 0.001) and postural stability in both the anterior‐posterior (*p* = 0.001) and medial‐lateral (*p* = 0.018) directions compared to the control group. Additionally, the findings of this study indicated enhanced lower extremity kinematics (*p* = 0.007) and improved ankle joint position sense during both dorsiflexion (*p* = 0.001) and plantarflexion (*p* = 0.020) in the intervention group relative to the control group.

**Conclusion:**

The findings of this study indicate that modified FIFA + 11 exercises enhance postural balance, proprioception, and ankle movement performance in Frisbee athletes. Consequently, these exercises can be utilised to prevent injuries and improve both postural balance and proprioceptive awareness, as well as the movement performance of Frisbee players' ankles.

**Level of Evidence:**

Level III.

**Abbreviations**
AJFAT: Ankle Joint Function Evaluation Questionnaire
COP: center of pressure
DKV: dynamic knee valgus
RSI: reactive strength index
SLDJ: single leg drop jump

## INTRODUCTION

Ultimate Frisbee, created in New Jersey in 1968, has grown into a global sport with around 7 million players and IOC recognition since 2015. Played at youth, collegiate, club and professional levels, it became a World Games medal event and saw the launch of the American Ultimate Disc League in 2012, now with 24 teams in the United States and Canada [28]. Its rapid expansion attracts athletes from diverse sports. Flying disc sports include games like Ultimate, Guts, Disc Golf and Decathlon [8], with Ultimate being the most popular. Ultimate is played on a 100 × 37 m field with 18 m end zones and involves two teams of up to seven players aiming to catch the disc in the opponent′s end zone. The sport is known for its fast pace, self‐officiating, and emphasis on fair play. Its physical demands—such as sprinting, quick direction changes, jumping, and diving—carry injury risks. Injury rates range from 48–68% [34], mostly occur in the lower limbs (72%), with running as the main cause (32%) [5]. Rates in Hong Kong are 5–6 times lower than in the United States, possibly due to differences in training, surfaces, or prevention methods.

Research on American professional athletes in 2020 showed that lower limb injuries most commonly involved the ankle (19%, *n* = 58), thigh (17%, *n* = 50) and knee (14%, *n* = 42) [15]. Lateral ankle sprains are a common injury in sports, accounting for approximately 85% of all ankle sprains. This type of injury has a high recurrence rate and can lead to persistent symptoms related to the injury [20]. Lateral ankle sprains may occur as isolated injuries or as part of a broader injury process that results in functional instability of the ankle [7]. Research indicates that individuals who suffer from ankle sprains often experience residual symptoms, such as pain, decreased proprioception, and impaired neuromuscular control, which increase the likelihood of re‐injury and chronic ankle instability [19]. Neuromuscular control exercises and strength training are integral components of rehabilitation for chronic ankle sprains, targeting muscle weakness and deficits in neuromuscular control. These interventions have also demonstrated effectiveness in enhancing balance and overall performance [22, 30]. Numerous studies indicate that balance and proprioceptive training significantly reduce the incidence and recurrence of ankle injuries across various sports populations, including football, basketball, and soccer. This benefit may also extend to players of ultimate Frisbee [18]. In many sports, athletes are required to perform a series of maximal vertical jumps repeatedly during competitions [29]. It is evident that superior jumping performance is directly linked to their success. Consequently, various exercises have been developed to enhance athletes' jumping performance, often by increasing muscle strength [33]. Additionally, as an observational motor screening test, jump‐land‐jump movement tasks have become increasingly valuable for identifying individuals with dysfunctional movement patterns, enabling targeted interventions as part of sports injury prevention programmes [2, 12].

A crucial aspect of injury prevention lies in athletes' awareness and adoption of preventive measures. The FIFA 11+ Injury Prevention Program, developed in 2006 by the FIFA Medical Assessment and Research Centre in partnership with the Oslo Sports Trauma Research Center and the Santa Monica Orthopedic and Sports Medicine Group, provides a structured warm‐up routine designed to reduce soccer injuries [3]. Its success has led to adaptations for other sports, with studies like David et al. demonstrating a 30% reduction in football injuries following its implementation [26]. However, research on the modified 11+ program′s impact on ankle injuries in Ultimate Frisbee remains scarce. This gap highlights the need for sport‐specific investigations to evaluate its effectiveness. While most studies on injury prevention utilising the FIFA 11+ program have been conducted in Western or East Asian populations, this study offers valuable insights by focusing on Iranian athletes. Thus, this study aims to assess the influence of an 8‐week FIFA 11+ training regimen on postural balance, proprioception, and movement performance in members of the Iranian National Frisbee team with functional ankle instability. We hypothesised that an 8‐week modified FIFA 11+ program would enhance postural balance, proprioception, and movement performance in Iranian national Ultimate Frisbee players with functional ankle instability.

## METHODS

### Participants

The current study is a prospective field experiment that investigates the effect of an independent variable, specifically the FIFA 11+ warm‐up training program, on dependent variables such as postural balance, proprioception, and movement performance. An a priori sample size calculation was conducted using G*Power version 3.1.0 (Franz Faul, University of Kiel, Germany) for an analysis of covariance (ANCOVA) with a single covariate. The calculation was based on a medium effect size (*f* = 0.25), an alpha level of 0.05, and a desired statistical power of 0.80. The results indicated a minimum requirement of 12 participants per group. To account for potential participant attrition, the sample size was increased to 26 individuals: An experimental group (*n* = 13) and a control group (*n* = 13). The primary investigator, a member of the Iranian national Frisbee team, was responsible for participant recruitment, data collection (including all protocols), and data analysis for both groups. The investigator conducted participant recruitment over a one‐month period, beginning on 10 September 2024. After recruitment, the experimental group completed the modified FIFA 11+ training program over eight weeks, while the control group continued their usual training. Participants were eligible for inclusion if they had been invited to the Iranian national Ultimate Frisbee team camp, were aged between 20 and 30 years, and had experienced functional ankle instability in the dominant foot for at least 12 weeks, as confirmed by an Ankle Joint Function Evaluation Questionnaire (AJFAT) score of 15–26. All participants were required to have been trained in Ultimate Frisbee at least twice per week and to be free from any underlying medical conditions that could affect performance or balance. Exclusion criteria included a history of lower limb fractures or surgeries, chronic ankle sprains affecting both ankles, use of medications influencing balance or neuromuscular function, receipt of physical therapy for the lower limb within the past 6 months, or the presence of neurological disorders. Participants were also excluded if they withdrew consent, missed training sessions during the intervention period, or sustained any acute ankle injury during the study.

### AJFAT

The AJFAT was utilised to assess ankle stability and function in individuals with functional ankle instability. The AJFAT demonstrated high test‐retest reliability (ICC = 0.94) and accuracy (SEM = 1.5). The questionnaire comprises 12 items addressing various aspects of ankle function, including pain, swelling, ability to walk on uneven surfaces, overall stability, strength, ability to descend stairs, running smoothness, change of direction while running, activity level, ability to sense and respond to ankle sprains, and ability to return to activity after sprains. Participants rated their ankle condition on a 5‐point scale (0 = much less than the other ankle; 4 = much more than the other ankle). The total score ranges from 0 to 48, with scores ≤ 26 indicating functional ankle instability. For this study, a score of 15–26 was considered indicative of instability [25].

### Pre‐ and post‐testing procedures

All participants underwent pre‐testing at the Allameh Tabataba'i University Sports Science Faculty laboratory. The pre‐testing included assessments of reactive strength, balance, two‐dimensional kinematic measurements of the lower limbs, and proprioception. Following the pre‐test, the experimental group performed the modified FIFA 11+ warm‐up protocol (for eight weeks, while the control group continued their routine team exercises. The primary investigator conducted a briefing session to explain the exercises to the coach and players and monitored the proper implementation of the protocol throughout the training period. Participants progressed to level three exercises. Post‐testing was conducted at the same laboratory after eight weeks. All pre‐ and post‐intervention tests were administered by the same examiner to ensure consistency and minimise examiner‐related bias.

### The FIFA 11+ protocol was performed twice weekly for 8 weeks and consisted of three sections

Section 1: Slow running combined with active stretching, controlled player collisions, correct landing techniques, and change of direction (8 min).

Section 2: Balance, strength, explosive power, and core muscle strengthening exercises, progressing from simple to advanced (10 min).

Section 3: Running exercises with high‐ and medium‐speed directional changes (2 minutes). Balance board exercises were added to Section 2 of the protocol [1, 31].

### Kinematic measurements

Markers were placed on each participant′s lower limb to approximate radiographic landmarks as described by Wilson et al. [36, 37]. The markers were positioned at the midpoint of the femoral condyles (knee joint center), the midpoint of the ankle malleolus (ankle joint center), and the proximal thigh along a line from the anterior superior iliac spine (ASIS) to the knee marker. Additional markers were placed on the ASIS. Frontal plane projection angle and hip adduction angle were calculated from video recordings of all participants during task performance. The knee pronation angle was measured as the angle between the thigh marker near the knee joint and the line from the knee joint to the ankle marker at the point of maximum knee bend. Positive values indicated knee valgus, while negative values indicated varus knee. The hip adduction angle was measured as the angle between the proximal thigh marker line and the line connecting the two ASIS [14].

### Data collection and analysis

#### Principal component analysis

##### Single leg drop jump (SLDJ)

Participants stood on a 30 cm platform with feet shoulder‐width apart and performed a forward jump to a marked spot (approximately half their height). Upon landing, they immediately executed a maximum vertical jump on the dominant leg. The dominant leg was determined as the leg used for single‐leg landing. The average of three repetitions was recorded [21].

### Two‐dimensional kinematic measurement of the lower limb

**Figure 1:**
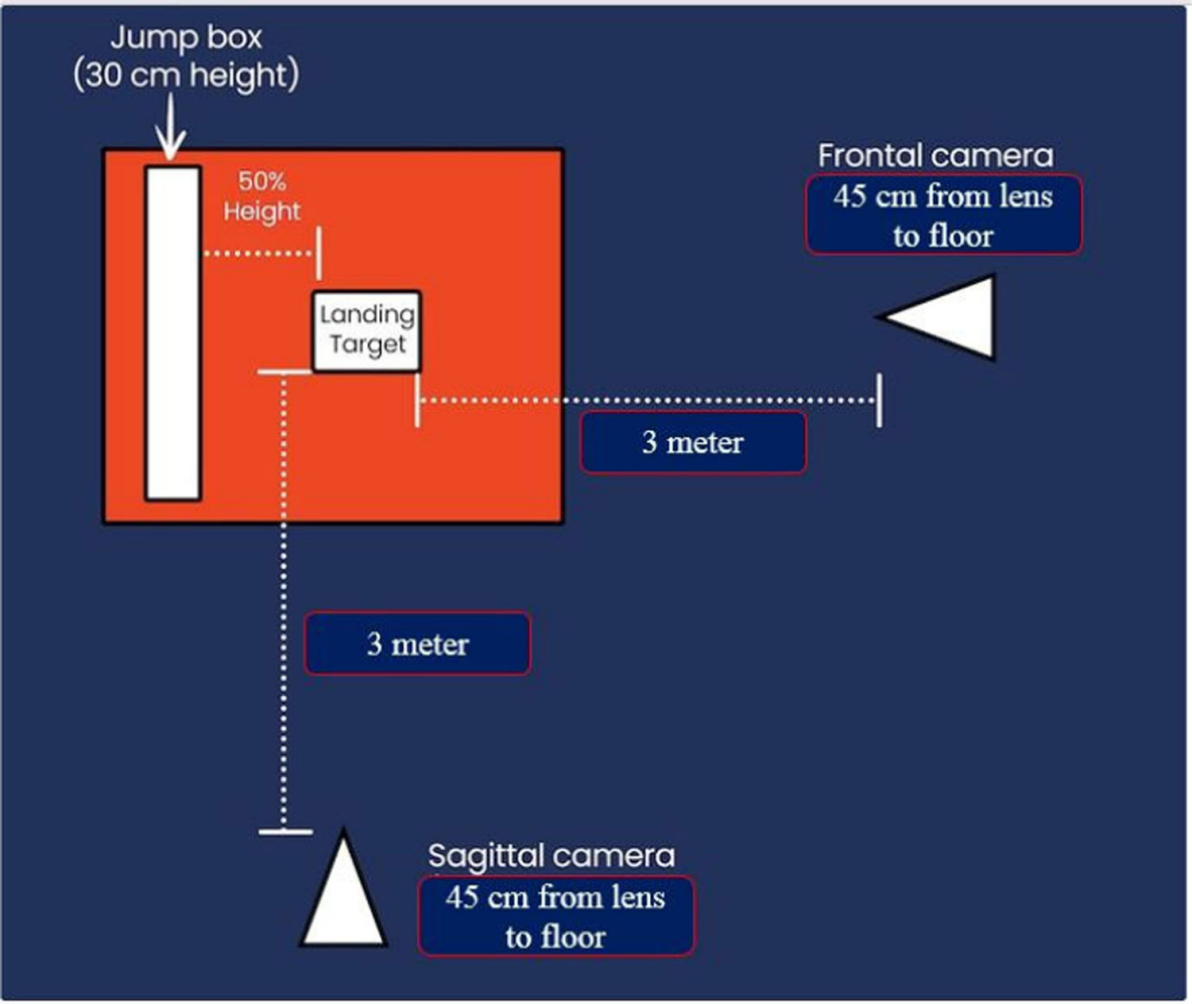
Schematic illustration of camera placement and marker setup during the single‐leg drop jump (SLDJ) kinematic assessment.

Knee joint angles in the sagittal and frontal planes were calculated at the moment of landing during the SLDJ task. Two video cameras (45 cm height, 3 m distance) recorded the movement from frontal and sagittal views (Figure 1). Kinova, an open‐access video analysis software, was used to analyse the recordings [17, 24]. The dynamic knee valgus index was calculated as the sum of two angles during the SLDJ task: (1) Knee valgus angle in the frontal plane (*β* angle) minus 180°, and (2) Hip angle in the frontal plane (*α* angle) minus 90°. Anatomical landmarks (e.g., ASIS, patella center and ankle center) were marked with coloured labels for accurate analysis [35].

### Reactive Strength Index (RSI)

The reactive power index was calculated by dividing the vertical jump height (calculated as (2 × flight time × 9.81)) by the ground contact time during the SLDJ test. Flight time was determined using a force plate′s force‐time diagram [23].

### Postural balance

Participants performed a single‐leg jump onto a force plate and maintained the position for 30 s. Center of pressure (COP) fluctuations in the medial‐lateral and anterior‐posterior directions were recorded. An average of three repetitions was used for analysis [10].

### Proprioception

Participants were seated with their legs hanging freely. They actively moved their ankles to a target position of 20° of plantar flexion, held it for 5 s, and then returned to a neutral position. After a 7‐s delay, they attempted to replicate the same angle with their eyes closed. The proprioception score was calculated as the average absolute difference between the target angle and the reconstructed angle across three repetitions.

### Statistical analysis

Descriptive statistics, including mean and standard deviation, were used to summarise demographic data, while the Shapiro‐Wilk test was conducted to evaluate data normality. Analysis of covariance (ANCOVA) was performed to determine the effect of the FIFA 11+ program on the parameters under investigation. Data analysis was conducted using SPSS version 26 (Microsoft Corp., Redmond, WA), with significance set at *p* ≤ 0.05.

## RESULTS

Subject characteristics are presented in Table 1. The control and experimental groups were comparable in terms of age, height, weight, and BMI (*p* > 0.05). The ANCOVA results revealed that the intervention group demonstrated significant improvements in jumping performance (*p* = 0.001) and postural stability in both the anterior‐posterior (*p* = 0.001) and medial‐lateral (*p* = 0.018) directions compared to the control group. Additionally, the findings of this study indicated enhanced lower extremity kinematics (*p* = 0.007) and improved ankle joint position sense during both dorsiflexion (*p* = 0.001) and plantarflexion (*p* = 0.020) in the intervention group relative to the control group (Table 2).

**Table 1:** Demographic characteristics of the subjects.^a^

Variables	Groups		
	Control (*n* = 13)	Experimental control (*n* = 13)	*p* value
Age, y	24.75 ± 2.79	24.25 ± 2.11	0.271
Wight, kg	64.59 ± 3.61	66.28 ± 4.37	0.249
Height, cm	173.59 ± 3.06	175.40 ± 4.46	0.381
BMI, kg/m^2^	21.37 ± 0.81	21.66 ± 0.82	0.620

*Note*: Significant level: *p* ≤ 0.05.

Abbreviations: BMI, body mass index; SD, standard deviation.

^a^Data are presented as mean ± SD.

**Table 2:** Evaluation of the postural balance, joint position sense, and dynamic knee valgus and reactive strength index during single‐legged drop jump task before and after interventions in both control and experimental groups.^a^

Variables	Groups					
		Control		Experimental		ANCOVA *p* value
		Pre‐test	Post‐test	Pre‐test	Post‐test	
Postural stability	Anterior‐posterior	4.29 ± 1.01	3.72 ± 1.50	3.40 ± 1.40	1.29 ± 1.43	0.001*
	Medial‐lateral	−2.71 ± 1.39	2.58 ± 0.65	−2.80 ± 1.46	1.95 ± 1.13	0.018*
Ankle joint position sense	Dorsiflexion	2.16 ± 0.94	2.27 ± 0.93	2.59 ± 1.01	1.17 ± 0.79	0.001*
	Plantarflexion	3.73 ± 2.87	3.60 ± 2.11	3.41 ± 2.63	2.05 ± 1.56	0.020*
DKV		18.37 ± 5.26	17.80 ± 5.22	15.47 ± 8.18	13.01 ± 6.73	0.007*
RSI		12.27 ± 3.94	12.25 ± 3.90	12.42 ± 2.99	18.32 ± 3.92	0.001*

*Note*: Conventional sign in postural stability: Medial‐lateral: − = non‐stance side, + = stance side; anterior‐posterior: − = posterior, + = anterior.

Abbreviations: DKV, dynamic knee valgus; RSI, reactive strength index; SD, standard deviation; SLDJ, single‐legged drop jump landing.

* Significant level: *p* ≤ 0.05.

^a^ Data are presented as mean ± SD.

## DISCUSSION

The findings of this study demonstrate that the 8‐week FIFA 11+ training program significantly improved dynamic balance, proprioception, and movement performance in Iranian national Ultimate Frisbee players with ankle functional instability. The analysis of covariance revealed a statistically significant difference between the training and control groups in post‐test measures of dynamic balance and proprioception. Specifically, dynamic balance improved in the post‐test compared to the pre‐test, and the joint angular reconstruction error decreased in the training group. These improvements can be attributed to the FIFA 11+ program′s emphasis on enhancing lower limb muscle strength, proprioception, and neuromuscular control, all of which are critical for postural stability and injury prevention. The program′s focus on muscle co‐contraction, core stability, and neuromuscular coordination likely contributed to these positive outcomes.

One of the key mechanisms underlying the improvement in dynamic balance is the increase in lower limb muscle strength [4, 6, 11]. Brito et al. reported that the FIFA 11+ exercises significantly enhance the strength of the muscles surrounding the knee, which aligns with the findings of this study [4]. Similarly, Hammami highlighted that increased lower limb muscle strength directly correlates with improved dynamic balance in athletes [13]. Furthermore, the FIFA 11+ program not only strengthens muscles but also enhances neuromuscular coordination, which plays a pivotal role in maintaining balance during dynamic movements. These combined effects likely explain the observed improvements in balance and proprioception among the participants. The inclusion of plyometric and neuromuscular training in the FIFA 11+ protocol appears to be a critical factor in improving proprioception and balance. Hubscher et al., in a review of balance and neuromuscular training, demonstrated that such training enhances neuromuscular communication and reduces the latency of proprioception, thereby decreasing the risk of acute injuries [16]. They also suggested that shifting proprioception from a feedback to a feedforward mechanism is an effective strategy for injury prevention. The current study supports this notion, as the FIFA 11+ program incorporates exercises such as single‐leg balance, plyometric jumps, and balance board activities, which likely contributed to the observed improvements in ankle proprioception and dynamic balance. For example, exercises like single‐leg squats on a balance board and vertical jumps in various directions challenge the sensorimotor system, promoting better proprioceptive awareness and control.

While the FIFA 11+ program has been widely studied in soccer players, its application to Ultimate Frisbee players is novel. Previous research has primarily focused on the program′s effects on knee joint proprioception [9, 27]. For instance, one study found that eight weeks of FIFA 11+ training significantly reduced proprioception error in the dominant leg at 45° and 60° but not at 30° or in the non‐dominant leg [1]. This suggests that the program′s effectiveness may vary depending on the joint angle and limb dominance, highlighting the need for sport‐specific adaptations. In the context of Ultimate Frisbee, the program′s emphasis on dynamic movements and unilateral exercises appears to be particularly beneficial for improving ankle stability and proprioception. The long‐term benefits of the FIFA 11+ program extend beyond balance and proprioception. A study on futsal players revealed that prolonged use of the program improved most biomechanical and physiological measures, although lower limb stability and ankle dorsiflexion showed limited improvements. This underscores the importance of tailoring the program to address specific weaknesses, such as ankle instability, which is a common issue in Ultimate Frisbee players. The sensorimotor system, which includes proprioceptive, visual, and vestibular components, plays a crucial role in maintaining balance and joint stability. By targeting these components, the FIFA 11+ program enhances overall functional stability and reduces injury risk.

The current study also evaluated the program′s impact on lower limb strength and stability indices. The results showed statistically significant improvements in both total stability and anteroposterior stability indices after eight weeks of training. These findings are consistent with those of Steffen et al., who reported that increased participation in the FIFA 11+ program led to improved functional balance in young soccer players [32]. The program′s focus on concentric and eccentric strength development, particularly in the hamstrings and quadriceps, likely contributed to these improvements. Additionally, the balanced ratio of knee flexor to extensor strength observed in this study suggests that the program effectively addresses muscle imbalances, which are a common risk factor for lower limb injuries.

We acknowledge that the current study has limitations that should be considered. First, the sample size was relatively small and limited to Iranian national‐level players, which may restrict the generalisability of the findings to other populations or skill levels. Another limitation is the absence of a long‐term follow‐up to assess whether the improvements in balance and proprioception are sustained over time or translate into reduced injury rates. Furthermore, the study did not control for external factors such as participants' training routines outside the FIFA 11+ program, which may have influenced the outcomes. Finally, the reliance on self‐reported measures for ankle functional instability and the lack of biomechanical analysis (e.g., force plate or motion capture data) may have introduced measurement bias. Addressing these limitations in future research would provide a more comprehensive understanding of the program′s efficacy and long‐term benefits.

## CONCLUSION

In conclusion, the FIFA 11+ program is a highly effective intervention for improving postural balance, proprioception, and movement performance in Ultimate Frisbee players with ankle functional instability. The program′s emphasis on strength, neuromuscular control, and sport‐specific exercises makes it a valuable tool for enhancing athletic performance and reducing injury risk. Future research should explore the long‐term effects of the program on injury rates and performance outcomes in Ultimate Frisbee players, as well as its applicability to other sports with similar demands.

## AUTHOR CONTRIBUTIONS

**Yeganeh Sharifi**: Scientific editing; writing–original draft preparation; investigation, methodology; supervision. **Farideh Babakhani**: Writing–original draft preparation; conceptualisation; methodology; data capture; data analysis. **Ramin Balouchy**: Methodology; writing–original draft preparation; investigation. **Mohamadreza Hatefi**: Conceptualisation; methodology; scientific editing.

## CONFLICT OF INTEREST STATEMENT

The authors declare no conflicts of interest.

## ETHICS STATEMENT

A study description was provided to all participants before the experiment, and all participants signed an informed written consent form. The study protocol received approval from the ethical committee of University in Iran. All methods were conducted in accordance with the relevant guidelines and regulations.

## Data Availability

The data sets utilised and/or analysed during the current study are available from the corresponding author upon reasonable request.
